# A MULTIDIMENSIONAL MODEL FOR POLYPHARMACY MEASUREMENT IN OLDER ADULTS: EVIDENCE FROM THE HEALTH RETIREMENT STUDY

**DOI:** 10.1093/geroni/igz038.2599

**Published:** 2019-11-08

**Authors:** Rebecca Bendayan, Ewan Carr, Alex D Federman, Richard J Dobson

**Affiliations:** 1 Dept. Biostatistics and Health Informatics, Institute of Psychiatry, Psychology & Neuroscience (IoPPN), King’s College London, London, United Kingdom, United Kingdom; 2 Division of General Internal Medicine, Department of Medicine, Icahn School of Medicine at Mount Sinai, New York, United States

## Abstract

Polypharmacy is associated with increased health care costs and adverse health outcomes. Traditional research on polypharmacy uses dichotomous measures which overlook its multidimensional nature. We propose a new approach to grouping older adults based on the number and type of medications taken as well as other indicators of polypharmacy. Data was extracted from 1328 respondents of the 2007 Prescription Drug Survey (a sub-study of the Health Retirement Study) who were between 50 and 70 years old and taking ≥1 medication each month. Latent class analysis was carried out with the optimal number of classes assessed based on relative model fit (AIC, adjusted BIC) and interpretability. Latent classes were formed based on the number of medications, drug types, duration of medication intake, side effects, and presence of chronic health conditions. A four-class model was selected based on model fit and interpretability of the solutions. Although there was some overlap when we compared our model with standard cut-offs for polypharmacy (i.e., ‘high polypharmacy’ classes were more likely to take 5+ and 9+ medications), chi-square tests showed significant differences between our latent classes and cut-offs based on 5+ [X2 = 894; p<0.001] and 9+ medications [X2 = 398; p<0.001]. Among individuals taking <5 medications, our model differentiated two distinct types of ‘low polypharmacy’ based on the types of drugs reported. Our proposal to incorporate a multidimensional assessment of polypharmacy considers the wider context of medication use and chronic health in older age, moving beyond crude medication counts.

